# Children With Autism Spectrum Disorder and Neurodevelopmental Regression Present a Severe Pattern After a Follow-Up at 24 Months

**DOI:** 10.3389/fpsyt.2021.644324

**Published:** 2021-03-26

**Authors:** Pilar Martin-Borreguero, Antonio Rafael Gómez-Fernández, Maria Jose De La Torre-Aguilar, Mercedes Gil-Campos, Katherine Flores-Rojas, Juan Luis Perez-Navero

**Affiliations:** ^1^Unit of Psychology and Paediatric Psychiatry, Reina Sofia University Hospital, Cordoba, Spain; ^2^Department of Paediatrics, Infanta Margarita Hospital, Cabra, Córdoba, Spain; ^3^Department of Paediatrics, Reina Sofia University Hospital, Córdoba University, Maimonides Biomedical Research Institute of Córdoba (IMIBIC), Córdoba, Spain; ^4^Centre for Biomedical Research on Rare Diseases (CIBERER), ISCIII, Madrid, Spain; ^5^Paediatric Research Unit, Reina Sofia University Hospital, Maimonides Biomedical Research Institute of Córdoba (IMIBIC), CIBERObn, Córdoba, Spain

**Keywords:** autism, children, neurodevelopmental regression, diagnostic measurements, autism severity

## Abstract

This study examined the presence of neurodevelopmental regression and its effects on the clinical manifestations and the severity of autism spectrum disorder (ASD) in a group of children with autism compared with those without neurodevelopmental regression at the time of initial classification and subsequently.

**Methods and Subjects:** ASD patients were classified into two subgroups, neurodevelopmental regressive (AMR) and non-regressive (ANMR), using a questionnaire based on the Autism Diagnostic Interview-Revised test. The severity of ASD and neurodevelopment were assessed with the *Childhood Autism Rating Scale Test-2, Strengths and Difficulties Questionnaire*, and *Pervasive Developmental Disorders Behavior Inventory Parent Ratings* (PDDBI) and with the Battelle Developmental Inventory tests at the beginning of the study and after 24 months of follow-up. Fifty-two patients aged 2–6 years with ASD were included. Nineteen were classified with AMR, and 33 were classified with ANMR.

**Results:** The AMR subgroup presented greater severity of autistic symptoms and higher autism scores. Additionally, they showed lower overall neurodevelopment. The AMR subgroup at 24 months had poorer scores on the Battelle Developmental Inventory test in the following areas: Total personal/social (*p* < 0.03), Total Motor (*p* < 0.04), Expressive (*p* < 0.01), and Battelle Total (*p* < 0.04). On the PDDBI test, the AMR subgroup had scores indicating significantly more severe ASD symptoms in the variables: ritual score (*p* < 0.038), social approach behaviors (*p* < 0.048), expressive language (*p* < 0.002), and autism score (*p* < 0.003).

**Conclusions:** ASD patients exhibited a set of different neurological phenotypes. The AMR and ANMR subgroups presented different clinical manifestations and prognoses in terms of the severity of autistic symptoms and neurodevelopment.

## Introduction

Autism and childhood disintegrative disorder (CDD) were classified as separate entities within pervasive developmental disorders (1) prior to the publication of the fifth edition of the *Diagnostic and Statistical Manual of Mental Disorders* (DSM) (2). CDD was characterized by a period of at least 2 years of apparently normal development, followed by a clinically significant loss of previously acquired skills (before 10 years of age) in at least two of the following areas: expressive and receptive language, social skills and adaptive behavior, bowel and bladder control, and play and motor skills. However, the fact that up to a third of patients with autism spectrum disorder (ASD) present neurodevelopmental regression (3), combined with the difficulty of identifying completely normal development during the first 2 years of life, has led to the disappearance of CDD as a diagnostic category, and it is now included within ASD.

The children with ASD exhibit a set of phenotypes including neurological alteration. Genetic components including gene mutation, copy-number variations, and epigenetic modifications play important and diverse roles in ASDs (4). One of the main problems where there is a notable lack of consensus is the establishment of criteria for determining regression. The most widely used criteria are those of the Autism Diagnostic Interview-Revised (ADI-R) test, while aspects related to the loss of language and social skills are the most widely studied and reviewed (5). The loss of language is undoubtedly the phenomenon that parents most frequently refer to when reporting the onset of symptoms (although at the time of regression, patients already tend to show a highly restricted linguistic repertoire), whereas the loss of motor or adaptive behaviors is much less common (5, 6). In terms of age, regression normally begins in the 2nd year of life, although it can emerge as late as 81 months (7). A review of 28 studies (4) reported that the mean age of the onset of regression was 20 months.

The significance of regression in clinical, pathogenic, and prognostic terms is insufficiently clear. Although regression has been previously linked to worse prognostic outcomes (8), such as impaired cognitive performance (9) and higher scores on the ADI-R (10), other studies have reported different findings (11). In a meta-analytic review (12), children with ASD and neurodevelopmental regression (AMR) were found to develop language skills before children with ASD and non-neurodevelopmental regression (ANMR) (3, 13). Studies based on homemade videos suggest that children with AMR exhibit higher levels of social and linguistic development than those with ANMR at the age of 1 year; but not at the age of 2 years (14), children with AMR apparently present a typical initial level of social development, unlike children with ANMR (15).

These results are not conclusive, and other authors suggest that typical development is rare in children with AMR (12, 14). The loss of previously acquired skills is not currently a diagnostic criterion, although some authors argue that it could be used as an early indicator of ASD (5), which could help to establish therapeutic strategies at an earlier stage. It is well-known that the ASD diagnosis is relatively stable over time, with up to 80% of adolescent and adult patients retaining an ASD diagnosis originally made during childhood (16).

Results of recent studies demonstrate that the onset of ASD involves alterations in the rates of key social and communication behaviors during the 1st years of life for most children. These and follow-up studies that extend past 36 months and continue evaluation of any child who presents with atypical early development and/or high-risk status suggest that regressive onset patterns occur much more frequently than previously recognized (17), with the consequence that the age of ASD diagnosis is often older than 4 years (18, 19).

The hypothesis of the present study is that children with ASD who undergo neurodevelopmental regression have a poorer prognosis than those who do not undergo regression, and this could help to establish earlier treatment strategies and care, thereby improving the long-term developmental outcomes. The main goal of this study was to classify a sample of children with ASD into AMR and ANMR subgroups, determine the severity of the ASD symptoms and the degree to which general development is affected, and assess the development of both subgroups 24 months after the start of the study.

## Methods

### Subjects

A longitudinal study was carried out on a cohort of children with ASD with broadly similar geographical and domestic conditions. The patients were diagnosed after being evaluated by two clinical psychologists specializing in ASD, who applied the DSM-5 criteria and various semi-structured clinical developmental interviews and psychological and behavioral tests that have been internationally recognized as reliable and valid for this purpose, primarily the Autism Diagnostic Observation Schedule-2 (ADOS-2) (13, 20). Before the patients were included in the study, two pediatricians specializing in developmental alterations and ASD conducted a systematic inspection and reviewed the medical history to rule out any other pathology associated with ASD.

This study was approved by the Hospital Biomedical Ethics Committee, and it conformed to the fundamental principles established in the 1964 Declaration of Helsinki. The patients with ASD were included in the study once they had been diagnosed and showed to fulfill the criteria for inclusion and exclusion, and after their parents or legal guardians had provided informed written consent.

The inclusion criteria were patients with ASD aged 2–6 years who had a positive score on the ADOS-2 test and fulfilled the DSM-5 criteria. Moreover, the parents and legal guardians agreed not to make any substantial changes to the psycho-educational treatment that the patients received during the monitoring period. The exclusion criteria were presence of another pathology associated with ASD, undergoing pharmacological treatment for any pathology or any comorbidity of ASD, taking food supplements or any alternative treatment, or the parents' or legal guardians' intention to modify the child's psycho-educational treatment in the following 24 months.

The group of ASD children was further divided into two subgroups based on presence or absence of neurodevelopmental regression during the first 2 years of life, which was assessed using a six-item questionnaire following the guidelines used by the ADI-R for the evaluation of this process (Table 1) (21). The ASD children who obtained a score equal to or >3 were included in the neurodevelopmental regression ASD subgroup (AMR), and those with a score <3 comprised the non-neurodevelopmental regression ASD subgroup (ANMR). The score 3 or >3 was considered a conservative cutoff point that would include in the AMR subgroup only the clearest cases of neurodevelopmental regression. The AMR subgroup presented neurodevelopmental delay, reaching a score lower than 70 on the cognitive quotient of the Battelle Developmental Inventory (BDI) test. All the children received developmental behavioral interventions.

**Table 1:** Questionnaire to classify autism spectrum disorder patients according to neurodevelopmental regression and non-neurodevelopmental regression.

**Questions**	**0 points**	**1 point**	**2 points**
1. Did your child at any time cease to say a word or words he/she had previously said?	**No**. He/she did not cease saying any words.	**Yes**. He/she stopped saying some words.	**Yes**. He/she stopped saying more than 10 words.
2. Did your child cease to make any gesture or imitation that he/she had previously made? (e.g., wave goodbye with their hand, play this little piggy, pretend to talk on the telephone).	**No**. He/she did not cease making gestures/imitations.	**Yes**. He/she stopped making the occasional gesture/imitation.	**Yes**. He/she stopped making various gestures or imitations.
3. Did your child at any time show reduced interest in other children? (e.g., stop approaching other children spontaneously, stop trying to communicate with other children…)	**No**. He/she did not show reduced interest.	**Yes**. He/she clearly showed partially reduced interest in other children.	**Yes**. He/she clearly showed reduced interest in other children.
4. Has your child ceased to play games he/she previously played? (e.g., playing ball, playing with cars appropriately, coloring in..)	**No**. He/she did not change his/her play.	**Yes**. He/she slightly changed his/her play.	**Yes**. He/she clearly changed his/her play.
5. Do you think that the symptoms appeared in a relatively sudden way?	**No**. The symptoms appeared gradually.	**Yes**. The symptoms appeared over several days or weeks.	**Yes**. The symptoms appeared suddenly.
6. Did the symptoms appear just after a specific event, like a bout of fever, gastroenteritis…?	No. The symptoms did not appear after a specific event.	Yes. The symptoms appeared not long after a specific event.	Yes. The symptoms appeared just after a specific event.

*Questionnaire based on the characteristics suggested for regression in the Autism Diagnostic Interview-Revised (ADI-R). Designed to collect information about neurodevelopmental regression in the first 24 months of life*.

### Standardized Diagnostic Measurements and Assessments of Autism Spectrum Disorder Severity

All the children with ASD underwent an initial developmental clinical interview, which identified the core symptoms of ASD, according to the DSM-5 clinical diagnostic criteria. Additionally, the following tests were administered in all the cases:

a) Autism Diagnostic Observation Schedule-2 (13, 20)
  Children were given an ADOS module consistent with their language development and age. It was administered by two clinical psychologists with official training in the administration and quantitative interpretation of this test for research purposes. All the children with ASD in the study exceeded the cutoff point for the diagnosis of ASD.
b) Pervasive Developmental Disorders Behavior Inventory Parent Ratings (22, 23)
  The standardized version of this test for the Spanish-speaking European population was used. This test was used to evaluate the symptomatic severity of pervasive developmental disorders in ASD patients. The Pervasive Developmental Disorders Behavior Inventory Parent Ratings (PDDBI), which was completed by all the parents of the children with ASD, evaluates the characteristic ASD core behavioral deficits (deficits in social interaction, language, and pragmatic communication and stereotyped behaviors), additional behavioral difficulties (fears and aggressive behaviors), and adaptive behaviors (social, linguistic, and learning skills). All the children with ASD in the study obtained a score ≥ 30.
c) Childhood Autism Rating Scale Test-2 (CARS-2) (24)
  This scale was designed to classify the severity of the autism pathology as mild, moderate, or severe.
d) Battelle Developmental Inventory, Second Edition (25)
  This test assesses the child's current level of development and functioning in five areas (personal/social, adaptive, motor movement, communication, and cognitive areas).
e) Strengths and Difficulties Questionnaire (26)
  This questionnaire is used to assess the presence of behavioral difficulties and adaptive behaviors.

### Statistical Analysis

With regard to the size of the ASD sample used in this study, given that the prevalence of ASD in Spain is calculated at 1% (27), data are expressed as mean ± SD (95% confidence intervals), median (IQR), or absolute (relative frequencies). Accepting an alpha risk 0.05 and a beta risk 0.2 in a two-sided test, 18 subjects in each subgroup were necessary to recognize a minimum difference in the variables selected as statistically significant. Common standard deviation was assumed to be 1.5. A 20% dropout rate was expected. The Shapiro–Wilk test was used for normally distributed data. Homogeneity of variances was estimated using Levene's test. The mean values for normally distributed continuous variables among subgroups were compared using the unpaired Student's *t*-test. The Mann–Whitney *U* test was applied for asymmetrically distributed data. Categorical variables were assessed using the chi-squared test or Fisher's exact test. Mixed-design ANOVA with Sidak correction was used to compare the BDI results of the AMR and ANMR subgroups at baseline and at the second administration (18–24 months later). Data were analyzed with the Statistical Package for the Social Sciences 22 (SPSS). All the tests were two-tailed, and a *p*-value < 0.05 was regarded as statistically significant.

## Results

Fifty-three patients with ASD were selected, and one was excluded on the grounds that neurodevelopmental regression could not be determined because the patient was an adoptee from another country and the family did not have the required information. No significant difference was found when comparing the average ages of the control group and the ASD group. When all the children were tested for regression, 19 fulfilled the criteria for AMR and 33 fulfilled the criteria for ANMR. The average age of the AMR subgroup (43.74 ± 11.91 months) was similar to the age of the ANMR subgroup (43.64 ± 10.747; *p* = 0.89). The sex ratio was similar for both subgroups (the AMR subgroup comprised 85% males, and the ANMR subgroup was made up of 81.8%; *p* = 0.55). Table 2 shows the percentage for each subgroup in relation to a questionnaire based on the characteristics for regression suggested in the ADI-R, the number and percentages of answers in the test.

**Table 2:** Number and percentages of answers on the test to classify children with autism spectrum disorder according to neurodevelopmental and non-neurodevelopmental regression.

**Questions**		**0 points**	**1 points**	**2 points**	***p***
1. Did your child at any time cease to say a word or words he/she had previously said?	ANMR	26 (78.8%)	7 (21.2%)	0	**<0.001**
	AMR	3 (15%)	14 (70%)	3 (15%)	
2. Did your child cease to make any gesture or imitation that he/she had previously made?	ANMR	26 (78.8%)	6 (18.2%)	1 (3%)	**<0.001**
	AMR	2 (10%)	9 (45%)	9 (45%)	
3. Did your child at any time show reduced interest in other children?	ANMR	30 (90.9%)	3 (9.1%)	0	**<0.001**
	AMR	8 (40%)	5 (20%)	7 (35%)	
4. Has your child ceased to play games he/she previously played?	ANMR	31 (93.9%)	2 (6.1%)	0)	**<0.001**
	AMR	7 (35%)	10 (50%)	3 (15%)	
5. Do you think that the symptoms appeared in a relatively sudden way?	ANMR	33 (100%)	0	0	**0.0001**
	AMR	7 (35%)	10 (50%)	3 (15%)	
6. Did the symptoms appear just after a specific event?	ANMR	33 (100%)	0	0	0.11
	AMR	15 (75%)	4 (20%)	1 (5%)	

*P < 0.05 value in bold, was considered for significant results*.

All the children had a score above the cutoff point for the diagnosis of ASD. No differences were observed in the scores for communication, interaction, play, and stereotypes on the ADOS test (results not shown). Cases scoring ADI-R ≥ 3 were classified as neurodevelopmental regression. ADI-R ≥ 3 was significantly related to the Childhood Autism Rating Scale Test (CARS), PDDBI, and BDI tests in the ASD subgroup that presented regression. The patients with ASD in the AMR subgroup had significantly poorer scores on the CARS test (AMR: 35.9 ± 8.12 vs. ANMR: 30.6 ± 6.11, *p* = 0.009), and significantly poorer autism scores on the PDDBI test (AMR: 53.53 ± 10.69 vs. ANMR: 46.22 ± 9.45, *p* = 0.022). On the BDI, the AMR subgroup had a lower overall score, indicating poorer neurodevelopment (AMR: 46.22 ± 9.45 vs. ANMR: 53.53 ± 10.69, *p* = 0.022).

In the detailed analysis of the various areas covered by the BDI (B. Total personal/social, B. Total Motor, B. Expressive and Battelle Total), significantly higher scores were observed in the ANMR subgroup compared with the AMR subgroup, which indicates greater global neurological development (Table 3). The PDDBI test results suggested that the ANMR subgroup was less severely affected in terms of social approach behaviors, expressive language, and autism score (Table 4).

**Table 3:** Comparisons of the Battelle Inventory results in the autism spectrum disorder subgroups with and without neurodevelopmental regression at baseline and at a follow-up of 24 months.

**Battelle inventory**		***N***	**Basal time**	***p*^**a**^**	**24 months**	***p*^**b**^**
B. Total personal/social	AMR	13	34.31 ± 9.97	**0.003**	37.08 ± 18.58	**0.03**
	ANMR	26	48.88 ± 14.84		51.58 ± 20.53	
B. Total adaptive	AMR	13	42.38 ± 11.59	**0.004**	40.69 ± 16.19	0.09
	ANMR	26	56.73 ± 14.52		51.38 ± 19.20	
B. Gross motor	AMR	12	64.58 ± 13.11	0.4	51.67 ± 17.24^*^	0.07
	ANMR	24	69.50 ± 17.77		66.46 ± 25.46	
B. Fine motor	AMR	12	58.83 ± 21.15	0.06	54 ± 19.31	0.06
	ANMR	24	71.08 ± 16.61		68.29 ± 22.2	
B. Total motor	AMR	13	62.23 ± 11.49	**0.05**	55.38 ± 18.39	**0.04**
	ANMR	26	71.31 ± 14.60		69.58 ± 21.17	
B. Receptive	AMR	12	32.92 ± 23.86	**0.01**	43.33 ± 25.51^*^	0.12
	ANMR	24	53.75 ± 21.35		57.29 ± 24.81	
B. Expressive	AMR	12	37.25 ± 14.75	**0.05**	42.08 ± 25.53	**0.01**
	ANMR	24	51.50 ± 21.9		55.33 ± 26.67	
B. Total communication	AMR	13	34.08 ± 16.79	**0.01**	43.77 ± 26.96^*^	0.17
	ANMR	26	50.50 ± 18.9		55.81 ± 24.68	
B. Total cognitive	AMR	13	62.08 ± 23.29	**0.03**	64.31 ± 26.47	0.31
	ANMR	26	78.12 ± 20.28		73.85 ± 27.96	
Battelle total	AMR	13	47.62 ± 11.28	**0.006**	48 ± 20.07	**0.04**
	ANMR	26	60.85 ± 14.35		62.92 ± 21.8	

*Mental Regression (AMR). Without Mental Regression (ANMR). The data are expressed as means ± DS. Mixed-Designs ANOVA with Sidak correction used to compare differences among the groups. P < 0.05 value in bold, was considered for significant results. p^a^. p between AMR and ANMR at baseline; p^b^: p between AMR and ANMR at 24 months of follow-up*;

* *p < 0.05 between baseline and 24 months of follow-up (intragroup)*.

**Table 4:** Analysis of the *Pervasive Developmental Disorders Behavior Inventory* test in the ASD subgroups with and without neurodevelopmental regression at baseline and at a follow-up at 24 months.

**PDDBI**	**Group**	***N***	**Basal time**	***p*^**a**^**	**24 months**	***p*^**b**^**
Sensory	AMR	16	52.63 ± 12.69	0.182	52.31 ± 10.49	**0.05**
	ANMR	27	49.22 ± 9.38		46.37 ± 9.36^*^	
Ritual score	AMR	16	52.88 ± 11.74	0.711	54.69 ± 9.66	**0.038**
	ANMR	27	52.56 ± 9.46		48.63 ± 8.52^*^	
Social pragmatic problems	AMR	16	49.13 ± 9.81	0.499	47.31 ± 7.19	0.257
	ANMR	26	46.73 ± 8.51		48.27 ± 6.75	
Semantic pragmatic problems	AMR	17	47.65 ± 9.95	0.315	48.94 ± 11.47	0.152
	ANMR	27	49.44 ± 9.32		49.33 ± 8.56	
Social approach behaviors	AMR	17	46.18 ± 9.47	**0.016**	50.12 ± 8.88^*^	**0.048**
	ANMR	27	54.04 ± 10.47		56.37 ± 10.48^*^	
Expressive language	AMR	17	46 ± 9.17	**0.006**	46.06 ± 11.14	**0.002**
	ANMR	27	55.3 ± 10.98		57.7 ± 11.23	
Score autism	AMR	17	53.53 ± 10.69	**0.022**	52.47 ± 9.73	**0.003**
	ANMR	27	46.22 ± 9.45		43.59 ± 8.67^*^	

*Analysis of the Pervasive Developmental Disorders Behavior Inventory test regarding the neurodevelopmental regression and non-neurodevelopmental regression groups at baseline and after follow-up at 24 months*.

*Mental Regression (AMR). Without Mental Regression (ANMR). The data are expressed as means ± DS. Mixed-Designs ANOVA with Sidak correction used to compare differences among groups. P < 0.05 value in bold, was considered for significant results. p^a^. p between AMR and ANMR at baseline; p^b^: p between AMR and ANMR at 24 months follow-up*;

* *p < 0.05 between baseline and 24 months follow-up (intragroup)*.

**Figure 1:**
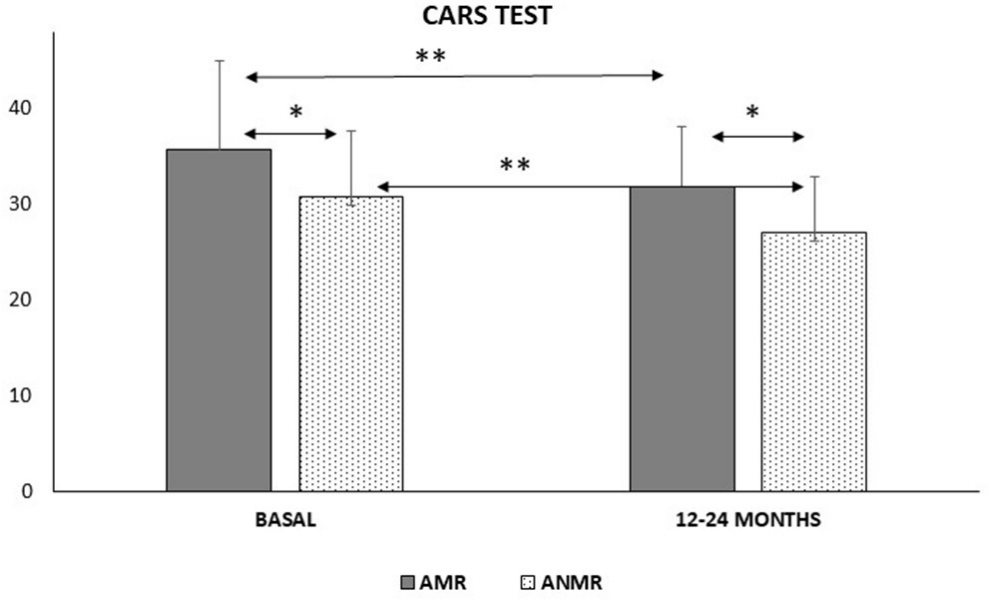
Comparison of the Childhood Autism Rating Scale Test (CARS-2) scores between the neurodevelopmental regression (AMR) and non-neurodevelopmental regression (ANMR) groups in two study times of follow-up declined similarly for both groups. *p < 0.05, **p < 0.01.

The Strengths and Difficulties Questionnaire (SDQ), CARS, BDI, and PDDBI tests were re-administered 24 months after the first assessment to evaluate how both subgroups had changed regarding general development. The SDQ test did not reveal any differences between the two dates. Scores on the CARS test declined similarly for both subgroups between baseline and at 12–18 months (Figure 1). The BDI exhibited improvements in the receptive and communication areas in the AMR subgroup, and the differences in these areas compared with the ANMR subgroup were eradicated as a result. However, the total motor movement skills of the AMR subgroup declined compared with the ANMR subgroup (Table 3). On the PDDBI test, the ANMR subgroup showed significant reductions in the sensory and autism scores (Table 4).

## Discussion

The results of the present study provide evidence of an ASD phenotype of neurodevelopmental regression during infancy, and how it differs from that of other groups without this symptomatology and severity, even after 24 months of follow-up. Thirty-five percent of all the children with ASD were classified as having neurodevelopmental regression using a especially designed questionnaire based on the regression characteristics suggested by the ADI-R. This result does not greatly differ from the figures reported by other authors (3) with other tools. All the ASD patients met the inclusion criteria. The data obtained from the tests, which were administered to the entire series, seem to indicate that the sample was correctly classified into two subgroups presenting symptoms of varying severity. Moreover, the questionnaire that was used to make this classification was designed following the criteria of the ADI-R clinical interview for regression (Table 1) and has been used by the current authors' research group with notable results (28, 29).

Initially, the AMR subgroup scored more poorly on all the tests assessing the presence or severity of ASD symptoms. On the BDI, which does not assess the presence of ASD symptoms but rather the child's overall development in such important areas as cognition, language, and adaptive behavior, the AMR subgroup also obtained worse results than the ANMR subgroup, with statistically significant differences in all the areas except gross motor movement. Therefore, the AMR subgroup seemed to exhibit not only more severe ASD symptoms but also general global development that was markedly poor, than did the ANMR subgroup. This result is consistent with the findings published by other authors (8–10).

The clinical evolution of both subgroups is significant. The data obtained after 24 months from the start of the study using the CARS, PDDBI, and BDI questionnaires show how the differences between the two subgroups persisted. The AMR and ANMR subgroups exhibited improvements in some areas of the Battelle, chiefly those related to language. In other areas, both subgroups presented changes, but they were not statistically significant. These findings suggest that the differences between the two subgroups in terms of global development were relatively stable after 24 months. As has already been noted, approximately 80% of patients who receive an ASD diagnosis in the 1st years of life continue to meet the diagnostic criteria in adolescence and adulthood (10). The data obtained from the present study, despite limitations stemming from the sample size and the fact that it only involved 24 months of monitoring, point to the possibility that the majority of the 20% of patients who cease to meet the ASD criteria in later stages of development may not belong to the AMR subgroup.

Ozonoff et al. (17–19) examined different ways of measuring the onset of symptoms of ASD. Their findings suggest that declining developmental skills, consistent with a regressive onset pattern, are common in children with ASD. Their results bring into question the accuracy of the methods used for measuring the onset of ASD symptoms. Thurm et al. (30) reviewed the evolution of autism diagnosis and ASD diagnostic tools. They considered the criteria for making the clinical distinction between intellectual disability (ID) with and without ASD. They assessed diagnostic boundaries between ID and secondary vs. idiopathic ASD. The diagnosis of ID in the context of an ASD may be one of the strongest indicators of an associated condition of secondary autism. In the fifth edition of the DSM, regression was not included as a diagnostic criterion (2). However, some authors have suggested the possibility of using neurodevelopmental regression as a precursor of the disorder (5), which would enable the diagnostic process to be streamlined and appropriate treatment to be started earlier. Prospective studies demonstrate that the onset of ASD involves deterioration in the rates of key social and communication behaviors during the 1st years of life for most children. The regressive onset patterns occur much more frequently than previously acknowledged (17).

In the present authors' context, the diagnosis of ASD involves a process that can often last months. Following the initial suspicions of some developmental alterations, a pediatrician working in primary health care is the first professional to assess the patient. Often, a lack of information and specific training in ASD can cause the pediatrician to adopt a conservative stance: cases are monitored for a few months to see how the patients develop before they are referred to a specialist clinic. Once they are referred to the appropriate clinic for assessment, not only is an initial clinical interview required but also specific tests for the diagnosis of ASD (generally the ADOS-2, in the case of the authors' hospital) must be administered.

Moreover, in the case of patients with high general ability, good language, and relatively intact non-verbal communication skills, it is only when they enter preschool, where they face increased behavioral expectations, particularly in terms of their responses to peers and unfamiliar adults, that parents, caregivers, and other professionals become concerned about possible atypical development (6). Consequently, diagnosis is delayed further still. As has been already noted, up to 20% of the children initially diagnosed with ASD cease to fulfill the diagnostic criteria in later years, which further complicate the establishment of a definite diagnosis in very young patients.

At present, early intervention programs are the only programs that have been found to improve behavioral alterations, aid neurodevelopment, and reduce the severity of ASD symptoms (31). In the present study, all the patients who were selected fulfilled the DSM-5 criteria for the diagnosis of ASD and scored above the cutoff threshold on the ADOS-2, with the AMR subgroup obtaining the highest scores. Therefore, although regression has not still been established as a diagnostic criterion, the authors agree with the view that has been expressed elsewhere that it could be used as a red flag to initiate early intervention immediately after regression, without needing to wait for a definitive diagnosis. Given the neural plasticity of young children, early intervention programs would be inducive of a better prognosis.

One of the most important benefits of determining a prognostic factor capable of identifying those patients who, at the time of diagnosis are at risk of developing unfavorably, is the ability to plan more aggressive treatment strategies. Currently, there are no curative treatments for ASD (32). Treatments that have exhibited greater efficacy in attenuating the most severe symptoms of the disorder have been intensive psycho-educational treatments. The presence of neurodevelopmental regression may indicate the need to begin this type of therapy in a timely way to maximize the developmental potential of the child. In addition to the use of neurodevelopmental regression as an isolated prognostic factor, it would be valuable to be able to link it to other well-established prognostic factors in ASD. Various authors have suggested a low cognitive level as an indicator of a poor prognosis of ASD.

In the present sample, the cognitive level, as determined by the BDI, was statistically significantly worse in the AMR subgroup than in the ANMR subgroup (AMR 61.72 ± 20.71 vs. ANMR 79.33 ± 18.89) (*p* = 0.021). On average, the AMR subgroup scored below 70, the point that indicates low functioning in ASD, and these differences were maintained in the second assessment 24 months later.

One interesting aspect for establishing the degree of ID is adaptive functioning (33–35). Indeed, in the DSM-5, adaptive functioning has replaced the intelligence quotient (IQ) as the means of classifying the severity of ID because what determines the degree of support that a person with ID needs is his or her degree of adaptation to the environment and not the IQ score determined with a psychometric test. In the present study, the BDI results show that the AMR subgroup had significantly worse adaptive functioning scores, a finding that persisted at the follow-up 24 months later. This finding suggests that, in principle, the AMR subgroup will require a greater degree of support to negotiate the demands of daily life than the ANMR subgroup, and that these differences may be maintained throughout childhood and probably into adolescence and adulthood. The children with ASD exhibited a degree of disability that fundamentally stems from the severity of their symptoms, although other factors, such as family support and the age of the patient, may also play a role.

The limitations of the present study include the difficulty of collecting a reasonably large sample of children with ASD drawn from similar geographical and family backgrounds. However, the number of children included was consistent with the estimated sample size and had sufficient power to detect differences and associations.

## Conclusions

The AMR and ANMR forms of ASD should be considered as different neurological phenotypes within ASD, with symptoms that are quantitatively different in their degrees of severity. Regressive onset patterns in ASD occur frequently; therefore, early detection of regression in neurodevelopment in these patients is a priority for establishing specific early strategies and individualized psycho-educational and pharmacological treatments.

## Data Availability Statement

The raw data supporting the conclusions of this article will be made available by the authors, without undue reservation.

## Ethics Statement

The studies involving human participants were reviewed and approved by Ethics committee of Investigation of Cordoba. Written informed consent to participate in this study was provided by the participants' legal guardian/next of kin.

## Author Contributions

PM-B, AG-F, and MD were involved in the experimental work. KF-R processed the experimental data and performed the biochemical analysis. MD realized the statistical analysis. PM-B, MG-C, MD, and JP-N drafted the manuscript. All authors participated in the discussion and final manuscript version and involved in the study design and plan work.

## Conflict of Interest

The authors declare that the research was conducted in the absence of any commercial or financial relationships that could be construed as a potential conflict of interest.

## References

[1] American Psychiatric Association (APA). Diagnostic and Statistical Manual of Mental Disorders DSM-IV-TR. Washington DC: American Psychiatric Association (2000).

[2] American Psychiatric Association (APA). Diagnostic and Statistical Manual of Mental disorders. 5th ed. Washington DC: American Psychiatric Association (2013). 10.1176/appi.books.9780890425596

[3] Baird G Charman T Pickles A Chandler S Loucas T Meldrum D. Regression, developmental trajectory and associated problems in disorders in the autism spectrum: the SNAP study. J Autism Dev Disord. (2008) 38:1827–36. 10.1007/s10803-008-0571-9 18449635

[4] Zhang XC Shu LQ Zhao XS Li XK. Autism spectrum disorders: autistic phenotypes and complicated mechanisms. World J Pediatr. (2019) 15:17–25. 10.1007/s12519-018-0210-2 30607884

[5] Al Backer NB. Developmental regression in autism spectrum disorder. Sudanese J Paediatr. (2015)15:21–6. PMC4949854 27493417

[6] Oslejsková H Dusek L Makovská Z Pejcochová J Autrata R Slapák I. Complicated relationship between autism with regression and epilepsy. Neuroendocrinol Lett. (|2008); 29:558–70. 18766162

[7] Stella J Mundy P Tuchman R. Social and nonsocial factors in the Childhood Autism Rating Scale. J Autism Dev Disord. (1999) 29:307–17. 10.1023/A:1022111419409 10478730

[8] Hansen RL Ozonoff S Krakowiak P Angkustsiri K Jones C Deprey LJ. Regression in autism: prevalence and associated factors in the CHARGE Study. Ambul Pediatr. (2008) 8:25–31. 10.1016/j.ambp.2007.08.006 18191778

[9] Wiggins LD Rice CE Baio J. Developmental regression in children with an autism spectrum disorder identified by a population-based surveillance system. Autism. (2009) 13:357–74. 10.1177/1362361309105662 19535466

[10] Meilleur AA Fombonne E. Regression of language and non-language skills in pervasive developmental disorders. J Intellect Disabil Res. (2009) 53:115–24. 10.1111/j.1365-2788.2008.01134.x 19054269

[11] Bradley CC Boan AD Cohen AP Charles JM Carpenter LA. Reported history of developmental regression and restricted, repetitive behaviours in children with autism spectrum disorders. J Dev Behav Pediatr. (2016) 37:451–6. 10.1097/DBP.0000000000000316 27366956

[12] Barger BD Campbell JM McDonough JD. Prevalence and onset of regression within autism spectrum disorders: a meta-analytic review. J Autism Dev Disord. (2013) 43:817–28. 10.1007/s10803-012-1621-x 22855372

[13] Lord C Shulman C DiLavore P. Regression and word loss in autistic spectrum disorders. J Child Psychol Psychiatry. (2004) 45:936–55. 10.1111/j.1469-7610.2004.t01-1-00287.x 15225337

[14] Werner E Dawson G Munson J Osterling J. Variation in early developmental course in autism and its relation with behavioural outcome at 3–4 years of age. J Autism Dev Disord. (2005) 35:337–50. 10.1007/s10803-005-3301-6 16119475

[15] Maestro S Muratori F Cesari A Pecini C Apicella F Stern D. A view to regressive autism through home movies. Is early development really normal? Acta Psychiatr Scand. (2006) 113:68–72. 10.1111/j.1600-0447.2005.00695.x 16390373

[16] Dos RSR Becker MM Ranzan J Bragatti MW Ohlweiler L. Follow up of patients with developmental delay and autistic spectrum disorders. Medicina. (2013) 73:16–9. 24072047

[17] Ozonoff S Iosif AM. Changing conceptualizations of regression: what prospective studies reveal about the onset of autism spectrum disorder. Neurosci Biobehav Rev. (2019) 100:296–304. 10.1016/j.neubiorev.2019.03.012 30885812 PMC6451681

[18] Ozonoff S Young GS Brian J Charman T Shephard E Solish A. Diagnosis of autism spectrum disorder after age 5 in children evaluated longitudinally since infancy. J Am Acad Child Adolesc Psychiatry. (2018) 57:849–57.e2. 10.1016/j.jaac.2018.06.022 30392626 PMC6235445

[19] Ozonoff S Gangi D Hanzel EP Hill A Hill MM Miller M. Onset patterns in autism: variation across informants, methods, and timing. Autism Res. (2018) 11:788–97. 10.1002/aur.1943 29524310 PMC5992045

[20] Pugliese CE Kenworthy L Bal VH Wallace GL Yerys BE Maddox BB. Replication and comparison of the newly proposed ADOS-2, module 4 algorithm in ASD without ID: a multi-site study. J Autism Dev Disord. (2015) 45:3919–31. 10.1007/s10803-015-2586-3 26385796 PMC4654671

[21] Kim SH Lord C. New autism diagnostic interview-revised algorithms for toddlers and young preschoolers from 12 to 47 months of age. J Autism Dev Disord. (2012) 42:82–93. 10.1007/s10803-011-1213-1 21384244

[22] Cohen IL Sudhalter V. PDD Behaviour Inventory (PDDBI). Lutz, FL: Psychological Assessment Resources (2005). Available online at: https://opwdd.ny.gov/opwdd_community_connections/autism_platform/parents_corner/the_pdd_behaviour_inventory (accessed October 2020).

[23] Cohen IL Schmidt-Lackner S Romanczyk R Sudhalter V. The PDD Behaviour Inventory: a rating scale for assessing response to intervention in children with pervasive developmental disorder. J Autism Dev Disord. (2003) 33:31–45. 10.1023/A:1022226403878 12708578

[24] Scholper E Van Bourgondien ME Wellman GJ Love SR. Childhood Autism Rating Scale- (CARS2). Los Angeles, CA: Western Psychological Services (2010). p. 658.

[25] Goldin RL Matson JL Beighley JS Jang J. Autism spectrum disorder severity as a predictor of Battelle Developmental Inventory - second edition (BDI-2) scores in toddlers. Dev Neurorehabil. (2014) 17:39–43. 10.3109/17518423.2013.839585 24088047

[26] Goodman A Goodman R. Strengths and Difficulties Questionnaire scores and mental health in looked after children. Br J Psychiatry. (2012) 200:426–7. 10.1192/bjp.bp.111.104380 22550331

[27] Morales-Hidalgo P Roigé-Castellví J Hernández-Martínez C Voltas N Canals J. Prevalence and characteristics of autism spectrum disorder among Spanish school-age children. J Autism Dev Disord. (2018) 48:3176–90. 10.1007/s10803-018-3581-2 29696527

[28] Gomez-Fernandez A de la Torre-Aguilar MJ Gil-Campos M Flores-Rojas K Cruz-Rico MD Martin-Borreguero P. Children with autism spectrum disorder with regression exhibit a different profile in plasma cytokines and adhesion molecules compared to children without such regression. Front Pediatr. (2018) 6:264. 10.3389/fped.2018.00264 30320048 PMC6169449

[29] Plaza-Díaz J Gómez-Fernández A Chueca N Torre-Aguilar MJDL Gil Á Perez-Navero JL. Autism Spectrum Disorder (ASD) with and without mental regression is associated with changes in the fecal microbiota. Nutrients. (2019) 11:337. 10.3390/nu11020337 30764497 PMC6412819

[30] Thurm A Farmer C Salzman E Lord C Bishop S. State of the field: differentiating intellectual disability from autism spectrum disorder. Front Psychiatry. (2019) 10:526. 10.3389/fpsyt.2019.00526 31417436 PMC6683759

[31] Tachibana Y Miyazaki C Ota E Mori R Hwang Y Kobayashi E. A systematic review and meta-analysis of comprehensive interventions for pre-school children with autism spectrum disorder (ASD). PLoS ONE. (2017) 12:e0186502. 10.1371/journal.pone.0186502 29211740 PMC5718481

[32] Stepanova E Dowling S Phelps M Findling RL. Pharmacotherapy of emotional and behavioural symptoms associated with autism spectrum disorder in children and adolescents. Dialogues Clin Neurosci. (2017) 19:395–402. 10.31887/DCNS.2017.19.4/rfindling 29398934 PMC5789216

[33] Pugliese CE Anthony L Strang JF Dudley K Wallace GL Kenworthy L. Increasing adaptive behaviour skill deficits from childhood to adolescence in autism spectrum disorder: role of executive function. J Autism Dev Disord. (2015) 45:1579–87. 10.1007/s10803-014-2309-1 25398602 PMC4433442

[34] Kenworthy L. Longitudinal examination of adaptive behaviour in autism spectrum disorders: Influence of executive function. J Autism Dev Disord. (2016) 46:467–77. 10.1007/s10803-015-2584-5 26349921 PMC4726475

[35] Golya N McIntyre LL. Variability in adaptive behaviour in young children with autism spectrum disorder. J Intellect Dev Disab. (2018) 43:102–11. 10.3109/13668250.2017.1287886 30581321 PMC6300052

